# Contribution of Sudanese medical diaspora to the healthcare delivery system in Sudan: exploring options and barriers

**DOI:** 10.1186/s12960-016-0123-x

**Published:** 2016-06-30

**Authors:** Fayrouz Mohammed Abdalla, Maye Abu Omar, Elsheikh Elsiddig Badr

**Affiliations:** School of Health Sciences, Ahfad University for Women, Arda Street, P.O. BOX 167, Omdurman, Sudan; Nuffield Centre for International Health and Development, University of Leeds, Leeds, UK; Sudan Medical Specialization Board, Khartoum, Sudan

**Keywords:** Diaspora options, Healthcare delivery system, Physicians, Migration, Sudan

## Abstract

**Background:**

Medical diaspora options, including the engagement of expatriate physicians in development efforts within their home country, are being called for to reverse the effects of brain drain from developing countries. This paper presents the results of a study exploring the potential contributions for the Sudanese Medial Diaspora Options to the healthcare delivery system (HCDS) in Sudan, focusing on the options of temporal and permanent returns and the likely obstacles faced in their implementation.

**Methods:**

This was a cross-sectional study using a mixed methods design including quantitative and qualitative approaches. For the quantitative approach, the study, which focused on the possible contribution of the diaspora to healthcare delivery in Sudan, was based on an online survey using random purposive and snowballing sampling techniques involving 153 Sudanese physicians working in Saudi Arabia and other Gulf States, the United Kingdom, the Republic of Ireland, and the United States of America. The qualitative approach involved in-depth interviews with returnee physicians and key informants in Sudan, focusing on the return experiences, the barriers for return, and the options to improve future contributions.

**Results:**

Despite contributions of the Sudanese medical diaspora being of a small scale considering the size of the phenomenon, as well as infrequent and not appropriately organized, their inputs to academia and the links built with overseas institutions and specialist clinical services were nevertheless remarkable. The main barrier to temporal return was inappropriate organization by the local counterparts, while those for permanent return of physicians were poor work environment, insufficient financial payment, unsecured accommodation, and offspring education. The study identified short-term return as a feasible option considering the country’s current conditions. Proper coordination mechanisms for short-term returns and facilitation of permanent return through stakeholders’ collaboration were proposed to improve diaspora contributions.

**Conclusions:**

The potentials of Sudanese medial diaspora contributions to the HCDS in Sudan are promising. Short-term contributions were observed as the best option for the current country situation. Creation of a coordinating body from within the healthcare sector in Sudan to effectively coordinate diaspora contributions is recommended.

## Background

Migration of physicians is a growing global phenomenon affecting the capacity of developing countries’ healthcare systems, especially in Africa, and compromising their ability to deliver equitable services [1, 2]. Sudan has been particularly affected by the migration of physicians [3]. The assessment of mechanisms to reverse the effects of migration in developing countries is now a global concern. The involvement of healthcare professionals in diasporas for the development of healthcare systems of source countries has been successfully implemented in several countries in collaboration with international organizations including the United Nations Educational, Scientific and Cultural Organization, the International Organization for Migration, and the United Nations Development Programme [4–6]. Diaspora, as defined by the International Organization for Migration, are populations of migrant origin within a given territory, developing multifarious links involving flows and exchanges of people and resources with the homeland [7]. Physicians living in diaspora regularly send remittances to their home countries, constituting a considerable portion of foreign revenue for many developing countries [8]. They also engage in development efforts in their home countries when they return, either permanently or temporarily, through initiatives such as skills transfer and capacity building activities. The collective skills, ideas and experiences of migrants make their repatriation represent a major advantage for the sustainable development of their home countries [4, 9].

**Fig. 1:**
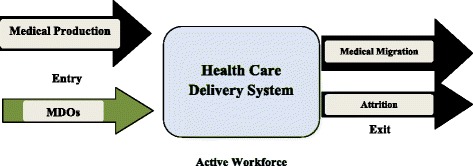
Physician’s dynamics in the healthcare delivery system in Sudan [10]

The World Health Organization’s framework Working Lifespan Approach to the Dynamics of the Health Workforce [10] is used herein to describe the dynamics of physicians’ migration from Sudan (Fig. 1).

The framework assesses three decision-making intersections, which have been adapted herein to the case of Sudan, including the magnitude, trends and causes of medical migration. Namely, (1) ‘entry’ into the healthcare delivery system (HCDS) [11],^1^ evaluating the number of graduate physicians, the production and employment policies, and the quality of medical education in Sudan; (2) the ‘active workforce’, covering the country’s responses to the medical migration phenomenon in terms of retention strategies and policies to stop migration; and, finally, (3) the ‘exit’ from HCDS, which includes medical migration as well as other causes of healthcare worker attrition such as retirement, career choice, and death.

Medical diaspora options (MDOs) are proposed as a strategy to help address the impact of medical migration. The present analysis includes a description of medical production (the entry), followed by the magnitude, trends and implications of medical migration (the exit), the country responses to medical migration in Sudan, and the MDOs as a proposed strategy.

### Medical production in Sudan

Medical education in Sudan has been provided since 1924, when such skills were in short supply globally, thus providing Sudanese physicians with international recognition and the privilege of being sought for employment overseas [3, 12]. In 1990, the country witnessed the start of a massive expansion in medical education, within both the public and private sectors, as a result of the so-called Revolution of Higher Education adopted by the government. The number of medical colleges increased from four in 1990 to 34 in 2012, resulting in a sharp increase of medical graduates, from 2499 in 1989 to 12,140 in 2008 [13, 14]. Female enrolment in medical education was relatively late, with only two students enrolled in 1946, but has since continued to increase; currently 56 % of registered medical students are female [14, 15].

The Ministry of Health is the main employer of physicians, with poor remuneration as compared to other African countries. For example, the entry salary of a physician was equivalent to only US$ 250 per month in 2010, compared to US$ 850 in Zimbabwe; when converted to Sudanese pounds, the figure becomes even more modest in view of the recent currency devaluation. This is in addition to the reported poor working conditions and the low absorptive capacity of the public sector in terms of long waiting lists for employment [13].

### Magnitude, trends and implications of medical migration in Sudan

Physicians’ migration in Sudan has dominated over other health professionals’ migration – since the early 1960s, Sudan has lost almost 60 % of its physicians to outmigration. It was estimated that approximately 12,000 out of 21,000 medical graduates registered in the Sudanese Medical Council in 2003 were practicing abroad [3]. About 3426 highly skilled Sudanese physicians with postgraduate qualifications in different medical disciplines are currently working and living abroad [6]. Further, the drain of physicians from Sudan continues, with a recent estimate showing that 30 % of the 3000 annual medical graduates migrate every year. These migrants are predominantly male, but a rising trend in female medical migration has also been observed recently [16].

**Fig. 2:**
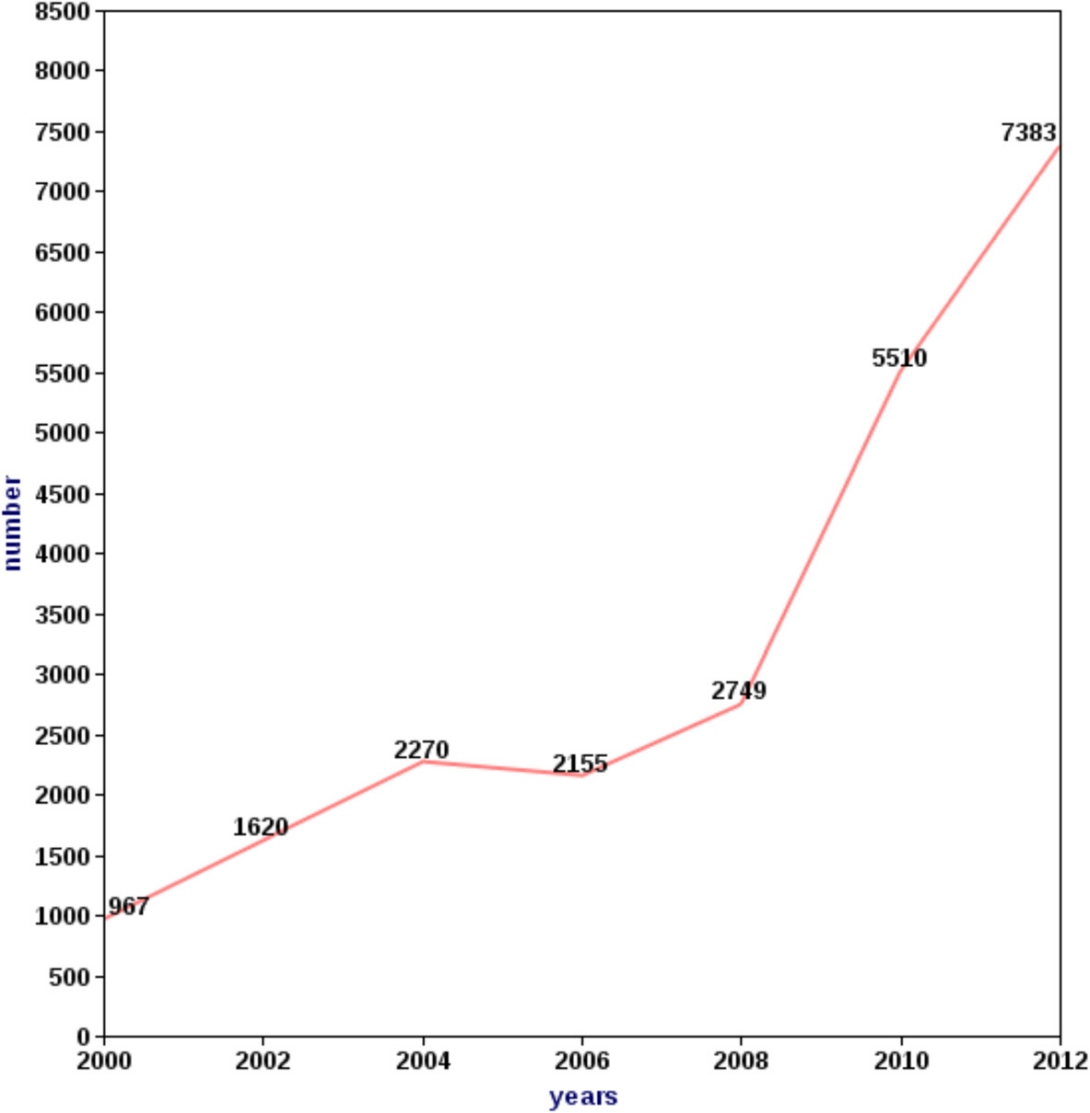
Trend of physicians obtaining experience certificate, 2000–2012, Sudan [13, 17]

Evidence, such as the number of physicians obtaining experience certificates, which are usually requested from the Ministry of Health to support job applications abroad, is used herein to indicate the magnitude of outmigration. The number of physicians requesting experience certificates has increased from 967 in 2000 to 7383 in 2012 [13, 17], indicating a rising trend in physician migration (Fig. 2) [13, 17].

Both general practitioners, non-specialized physicians with 3 years of experience after graduation, and specialists, awarded subspecialty degrees in one major or minor discipline of medicine and accredited from appropriate boards, are migrating from Sudan, although migration is relatively higher among the former [13]. The causes of medical outmigration are mostly financial, coupled with a lack of advanced training and career development opportunities, both of which are perceived as important for general practitioners in Sudan [3, 18]. The recent active recruitment of physicians, especially general practitioners, from Sudan to work abroad has been a driving force for outmigration – over 4000 physicians were documented to have registered in licensed recruitment agencies in 2010 [16]. The main destinations for Sudanese physicians are, respectively, Saudi Arabia, Gulf States, the United Kingdom, the Republic of Ireland, and the United States of America [3, 16].

This massive outflow of physicians from Sudan has had a negative impact on the quantity and quality of healthcare services in the country. Shortages in physicians are reported from many states in the country [19] due to either the migration of specialized physicians or that of postgraduate training teachers in some critically needed specializations [20]. Moreover, migration of experienced and senior staff has affected the quality of services in rural hospitals due to poor training and lack of supervision [3].

### Country responses to migration

The government of Sudan has exerted many efforts to stop medical migration. The introduction of a compulsory 1-year national service for physicians after completion of their internship was used as a safeguard strategy against immediate migration after graduation, by making completion of the national service a pre-condition for physicians to obtain their university qualification [21]. This was coupled with a policy restricting physicians’ overseas travel without written approval from their employer [22].

Efforts to retain physicians through provision of monetary and non-monetary incentives were attempted in Sudan, including provision of generous salaries, leading positions, housing, transport, and free university education for their offspring. However, these efforts were limited to highly specialized physicians in the formal sector and, due to the scarcity of resources, it could neither be generalized to all physicians nor sustained in the longer term [3].

Considering the financial difficulties of developing countries and the tempting offers abroad, health professional migration from developing countries is not likely to stop in the near future [23]. Developing countries, therefore, are advised to focus their efforts on managing migration rather than attempting to stop it [8, 23].

Migration of health professionals has long been studied from the perspective of loss (brain drain), focusing on the negative implications on healthcare systems of the source countries. Recently, some evidence from transnational migration theories has considered a wide range of possible contributions of immigrants to the development of their home countries, including MDOs [24].

### Medical Diaspora Options (MDOs)

Sudanese medical diasporas are well connected and linked through web-based society groups and have organized many initiatives to contribute to the HCDS in Sudan [3]. Many successful initiatives by visiting Sudanese consultants have been documented [13], as well as programs and voluntary contributions funded by international organizations such as the United Nations Development Programme (TOKTEN Project) [25] and expatriate professional associations such as the Sudan HIV/AIDS Working Group (SHAWG), a voluntary network of Sudanese expatriate healthcare professionals with special interest in HIV/AIDS which has contributed to the improvement of the quality of care provided to HIV/AIDS patients in Sudan [6].

Development of MDOs could address the impact of medical migration on the HCDS in Sudan. This paper explores the potential for the Sudanese medial diaspora to contribute to the HCDS in Sudan by examining MDOs focusing on engagement initiatives through temporal or short-term return and/or permanent return. Feasible and practical MDOs in Sudan’s context and the obstacles facing these MDOs are discussed herein. The paper specifically aims to examine Sudanese medical diaspora contributions to the HCDS in Sudan, assessing the views of Sudanese physicians living abroad and of those who have permanently returned regarding their contribution to the HCDS, identifying barriers to the MDOs in Sudan, and examining stakeholders views on options for effective, feasible and practical involvement of the Sudanese medical diaspora in the HCDS in Sudan.

## Methods

**Fig. 3:**
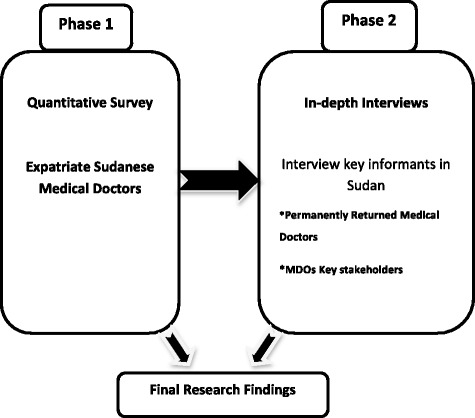
Mixed methods approach: phases of data collection

This was a cross-sectional study that was carried out in two separate phases. It used a mixed methods that combined both quantitative and qualitative approaches asillustrated in Fig. 3.

The study was based in Sudan and involved specialized and non-specialized (general practitioners) Sudanese physicians resident in Saudi Arabia, Gulf States, the United Kingdom, the Republic of Ireland, and the United States of America, which are the main destination countries for Sudanese physicians [3, 26], as well as permanently returned physicians and policy and decision makers from the human resources for healthcare stakeholders involved in medical diaspora contributions in Sudan such as the Federal Ministry of Health, the Ministry of Higher Education, the Secretariat for Sudanese Working Abroad, and national medical professional bodies.

Data were collected from June to November 2012. The use of mixed methods presented opportunities ranging from strengthening of the research design to verifying the research findings [27]. The quantitative approach used in the first phase of this study helped in designing the instruments used for the qualitative approach in the second phase. The findings from the survey fostered new lines of thinking included in the in-depth interviews and provided an opportunity for triangulation and cross data validation [28, 29].

### Phase one: quantitative survey

A survey was used to quantify the views of Sudanese physicians living in the United Kingdom, Republic of Ireland, Gulf States, and the United States of America at the time of data collection, so as to establish the most common type of contribution as well as to identify the most frequent barriers facing diaspora short-term contributions.

There were no available records of individual contact information [7], making the use of probability sampling impossible due to the absence of a sample frame to enable random sampling [30]. Accordingly, two sampling strategies were used to select participants. Random purposive sampling was used to provide the diversity required to examine different opinions and views [31]. Firstly, the existence of internet-based medical diaspora networks, associations and professional groups in addition to their social networks were used to distribute the electronic questionnaire, which utilized Bristol Online Survey software [32]. The questionnaire contained close ended and some open-ended questions to incite a wider range of responses while enabling statistical analysis [29, 33]. Secondly, a snowball sampling technique was used to increase the sample size [31]. Physicians registered in the abovementioned networks and associations were asked to resend the questionnaire link to other physicians they may know through email.

The sample size used in this study corresponded to the survey response rate [34]. The target sample was calculated based on Yamene’s sample size formula [35] as 400 participants, considering the approximate total population of 21,000 (estimated total number of physicians working outside Sudan) [26], using a ±5 % precision level. The response rate was 38 % (153 out of 400), which was higher than the overall stated level of success rate of 20 % [36].

### Phase two: in-depth interviewing

In order to understand the context in which decisions to contribute were made and subsequently enacted, it was necessary to understand how the returnees with previous diaspora engagement experiences, as thinking agents, selected types of contributions and positioned themselves within the HCDS in Sudan. Questions which probe the decision making of the returnees and the meaning of their actions can best be addressed through the use of qualitative techniques such as in-depth interviews [27, 33]. Interviews were conducted using a semi-structured interview guide. Respondents included managers and decision makers involved in the management of medical diaspora contributions in Sudan. They also included physicians who had permanently returned from abroad. Participants were interviewed in Arabic to enhance communication and all interviews were audio recorded following respondents’ permission to preserve the exact answers in addition to handwritten notes [37].

Participants for in-depth interviews were selected using purposive sampling of qualitative methods [38]. A total of 17 in-depth interviews were conducted, 10 with the key informants in Sudan and seven with physicians returned from abroad. The interviews were stopped when a point of data saturation was reached [39].

### Data analysis

Data collected through the survey questionnaire were analysed using Bristol Online Survey analysis tools for both close and open-ended questions [32]. The analysis of data from the qualitative interviews was performed following the general outlines of thematic analysis for qualitative data [40].

### Quality assurance

Measures to ensure validity and reliability were considered using design triangulation where different data sources and methods were used [29]. Thorough descriptions of the context and the use of quotations were employed to reflect more realistic results [29, 41]. Translation and back translation of transcripts from Arabic to English to Arabic was performed to ensure the quality of translation [42]. All procedures in research implementation, including research methods and approaches, were documented using a methodological log [43]. The study fulfilled the recommendations for the Federal Ministry of Health and the Sudan Medical Specialization Board ethical committees. Confidentiality of participants was protected during data collection, analysis and results dissemination.

### Study challenges and limitations

Absence of a sample frame was a limitation for having a probability sampling. This limitation was overcome by the use of purposive sampling. Challenges that resulted from self-reporting of respondents, such as incomplete filling of the questionnaire, were overcome by making the response of general questions a mandatory requisite to proceed to the following questions through the use of special setting options in the surveying software.

As the sample of the quantitative part depended mainly on the response rate, strategies to increase the response rate were applied, such as contacting the directors of diaspora medical associations and group modulators to facilitate questionnaire dissemination, sending invitation letters to participants, designing the questionnaire to include a reasonable number of questions requiring just 15 to 20 min to complete, and testing of the questionnaire in a small group for its content and ease of online use, in addition to designing a schedule reminder and specifying an end date for responses [34].

## Results

### Findings of phase one: the quantitative survey

The quantitative results were not aimed to test any hypothesis but rather to examine the group experiences of Sudanese physicians working aboard.

The study involved 153 respondents, all physicians working outside Sudan at the time of data collection. Out of the 153 respondents, 72 % were aged 30–50 years and 82 % of the respondents were male. Overall, 83 % of the respondents said that they acquired additional academic/professional qualifications while working abroad. Further, 44 % graduated between 1991 and 2000 and approximately 57 % had worked in Sudan for less than 5 years before migrating. The characteristics of respondents who took part in the first phase of the study are shown in Table 1.

**Table 1:** Characteristics of the participants in phase one (*n* = 153)

	Number	Percentage
Age, years		
<30	6	4 %
30–40	58	38 %
41–50	52	34 %
51–60	34	22 %
>60	3	2 %
Gender		
Male	125	82 %
Female	28	18 %
Year of obtaining medical qualification		
Before 1980	5	3 %
1980–1990	40	26 %
1991–2000	67	44 %
After 2000	41	27 %
Number of years worked in Sudan before migration		
<5	87	57 %
5–10	50	33 %
11–15	8	5 %
>15	2	1 %
Not worked in Sudan before	6	4 %
Additional academic qualification acquired after migration		
Physicians earned additional academic qualifications after migration	127	83 %
Physicians did not earn additional academic qualifications after migration	26	17 %

The majority of respondents (76 %) expressed a willingness to contribute to the HCDS in Sudan (Table 2), while 58 % of the respondents reported to have contributed to the HCDS in Sudan in the past. Among those who disagreed to contribute to the HCDS in Sudan, 48 % stated a lack of support from the government as the main reason. Other reasons reported were ineffectiveness of contributions, lack of appreciation of their contributions and insufficient organization of contributions by diaspora associations, in addition to political disagreements with the governing regime. Most respondents’ contributions were to clinical services and academia, representing 35 and 31 %, respectively. Other contributions included monetary donations, management consultancies, and advocacy for health, which were reported by 16, 10 and 8 % of respondents, respectively.

**Table 2:** Participant views in phase one regarding the contribution to the healthcare delivery system in Sudan (*n* = 153)

Statement	Strongly agree, *n* (%)	Somewhat agree, *n* (%)	Neither agree or disagree, *n* (%)	Somewhat disagree, *n* (%)	Strongly disagree, *n* (%)	Don’t know, *n* (%)
I am willing to contribute to Sudan’s HCDS from abroad	69 (45)	47 (31)	13 (8.5)	13 (8.5)	2 (1)	9 (6)
I intend to return to Sudan to live and work there	64 (42)	28 (18)	18 (12)	8 (5)	15 (10)	20 (13)

**Fig. 4:**
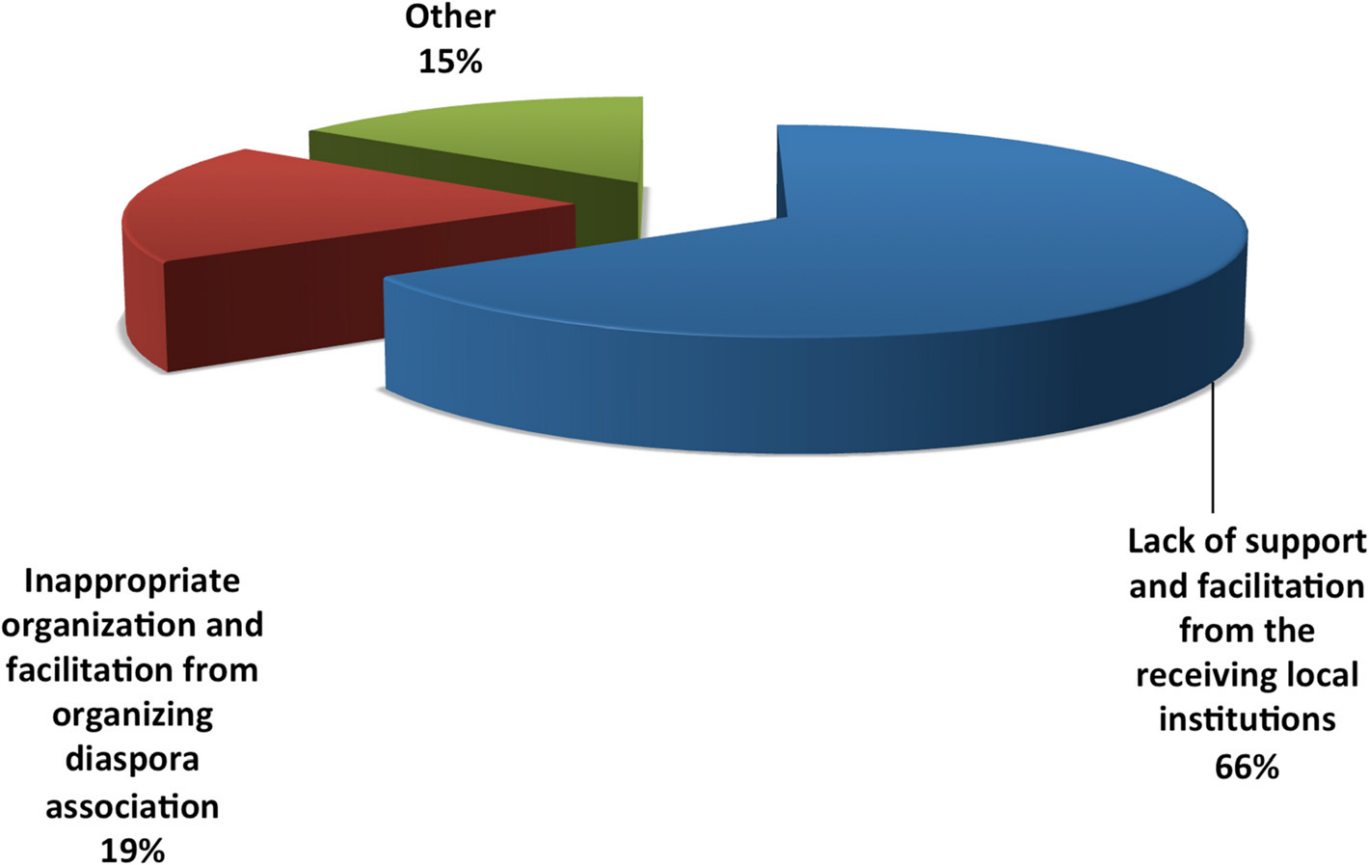
Challenges that faced the respondents’ previous contributions to the healthcare delivery system in Sudan (*n* = 89)

Overall, 44 % of responders stated that their contributions were personally initiated, whereas the diaspora professional associations and the government of Sudan had only organized 25 and 21 % of respondents’ previous contributions, respectively (Table 3). A lack of support and facilitation from the receiving local institutions was reported by 66 % of respondents as a challenge to their previous contributions (Fig. 4).

**Table 3:** Organizers of the previous medical diaspora contributions to the healthcare delivery system in Sudan (*n* = 89)

Organizer	Number	Percentage
Personal initiative	39	44 %
Diaspora professional association	22	25 %
Government of Sudan	19	21 %
Family and friends	9	10 %
Total	89	100 %

Although 61 % of respondents stated that their families were well settled in their adoptive country, the same percentage of respondents (60 %) planned to permanently return to Sudan at some point in their lives (Table 2). On the other hand, 36 % of respondents were unable to determine the exact dates for their return, giving a response of ‘I don’t know’, whereas 31 % answered that they would return in less than 5 years and 19 % when economic and political conditions allowed. Notably, all respondents had an intention to return, with none selecting the response ‘will never return to Sudan’ (Table 4).

**Table 4:** Time planned to permanently return to Sudan and the place of work in Sudan (*n* = 153)

Statement	Less than 5 years, *n* (%)	5–10 years, *n* (%)	When economic and political conditions permit, *n* (%)	Will never return to Sudan, *n* (%)	Don’t know	
Time planned to permanently return to Sudan	47 (31)	22 (14)	29 (19)	0 (0)	55 (36)	
Place of intended work when return to Sudan	Public hospital	Private hospital	Public university	Private university	Own private work	Others^a^
	46 (30)	3 (2)	40 (26)	7 (5)	44 (29)	13 (8)

^a^Others: dual practice in both public and private sectors

Over half of the respondents (56 %) mentioned that, on their return, they intended to work primarily in the public sector, i.e. public hospitals (30 %) and public universities (26 %). The remaining 29 % wanted to have their own private practice, 2 % would work in the private sector, 2 % in private hospitals, 5 % in private universities, and 9 % would have a dual practice in both public and private sectors (Table 4).

### Findings of phase two: qualitative in-depth interviews

The interviews of returnees and stakeholders of diaspora contribution elicited the following impressions and views. Consideration was given to the description of return experiences and circumstances, as well as to integration experiences within the HCDS in Sudan in order to understand different dimensions of the phenomenon. The themes that were explored emerged primarily from the results of the quantitative survey.

### Stakeholders’ opinion on the usefulness of the medical diaspora contributions to the HCDS in Sudan

Most of the stakeholders considered the contributions of the Sudanese medical diaspora to be of a relatively small magnitude in comparison to the number and expertise of the expatriate Sudanese physicians. Nevertheless, the contribution of physicians in diaspora was greater than that of the permanent returns, which was becoming rare due to recent financial constraints. The contribution of those who temporarily returned to Sudan was reported to be useful, especially in the fields of academia, knowledge and skill transfer, and in the establishment of international institutional links as shown in the following stakeholder participant statements:

“*Expatriates facilitated agreements and development of Memorandums of Understanding for collaboration in training.*”

“*We are now connected to internationally well-known universities through links with expatriates.*”

When it comes to remote parts of the country, their contributions were to a large extent concentrated to their areas of origin, as stated by one key informant from the Federal Ministry of Health:

“*Some contributed to rural and remote areas in Sudan to provide services in their original places through mainly personal efforts.*”

Recruitment of physicians from diaspora was mainly influenced by personal relations. The majority of academic institutions from both public and private sectors recruited expatriate physicians, whether for temporal or permanent jobs, based on personal links and contact networks as stated by one key informant from an academic institution:

“*We usually contact expatriates through personal links through professional networks and associations and through our alumni networks.*”

### Stakeholders’ opinion on appropriate, feasible and practical diaspora options for Sudan

The majority of the stakeholders considered short-term contributions as the most appropriate option for Sudan, considering their size and the current economic situation, as shown in the following quotes:

“*The number of the permanent returns is not that big … not as contributions through temporal returns which are generally big*”*.*

“*I think the country should focus on means to improve expatriate contributions mainly through short-term visits… because with the recent economic hardship permanent return is not an appropriate option*”*.*

### Stakeholders’ opinion on options to improve future contributions to the HCDS in Sudan

Most of the suggestions made by the key informants to improve medical diaspora contributions focused on the issue of coordination of contributions from both inside and outside of Sudan.

A body to coordinate contributions of the expatriate Sudanese physicians to the HCDS in Sudan was suggested by the majority of the key informants of the study. The suggested areas for coordination included types of contributions to academic institutions and to the clinical services. The proposed body was suggested to include representatives of different clinical specialities in order to make use of diaspora contributions to fill the needs and gaps in those particular fields.

“*I suggest every medical specialization to form an executive body to coordinate contributions of the expatriate specialists … To analyze their capacities and to decide the best way to be effectively used for the country welfare*”*.*

However, the issue of medical diaspora representation was raised by some of the key informants and a body to represent medical diaspora from outside Sudan was suggested to cater for the diversity in medical diaspora representations as stated in the following quote:

“*I suggest a recognized body representing expatriate Sudanese physicians… Because there are many associations and bodies of various political orientation, specializations in different host countries with different circumstances…*”

### Returnees’ experience of their contribution before permanent return to Sudan

All contributions were reported to be sporadic, irregular and with no long-term arrangements. Contributions to the public sector were common for temporal returns, where the majority of returnees reported to have provided support to the public sector, ranging from academic activities in universities to service provision and consultancies. The main reason given for the temporal contribution to the public sector was that the returnees felt that is where it was needed the most.

Host countries’ policies towards diaspora contributions were reported to determine both the degree and type of temporal contributions. Returnees from Saudi Arabia and Gulf States reported supportive policies towards contribution through associations, groups and individuals. Returnees from the United Kingdom and the Republic of Ireland reported an absence of hindrances and a neutral environment, whilst returnees from The United States of America reported contributions in academic gatherings and difficulties in arrangements of group contributions and physical donations. Most of the contributions were initiated by diaspora members and took place during their annual leave when they visited Sudan. The majority of contributions were voluntary in nature, involving academic contributions, which are usually organized through personal links. They contributed to teaching in universities, attendance of scientific gatherings and presentations in conferences. Other contributions were physical and monetary donations to public facilities by returnees from Saudi Arabia and other Gulf States.

Barriers to diaspora contributions were reported to be mainly experienced in Sudan rather than in the host countries. The absence of a focal point or organizing body responsible for coordination of contributions was reported to be the main barrier to diaspora contributions for almost all returnees, as exemplified by one of the returned physicians:

“*We did not know where to go or whom to contact*”*.*

The dual residency card issued by the Ministry of Interior in Sudan was considered by one of the returnees as a facilitating tool for contribution of expatriates with double nationalities as stated in the following quote:

“*For people like me who have double nationalities, this card facilitates entrance to and departure from Sudan… It is a very good facilitator because it allows people to maintain their foreign nationality while facilitating their residence in Sudan*”*.*

### Reasons for permanent return to Sudan

In most of the narratives on the causes of return to Sudan, the main direct factors were associated with social and family reasons. Having offspring in higher education was a major factor expressed by most returnees from the five research areas as well as other social reasons such as elderly parent care. However, the original intention to return was considered by the majority of returnees as the drive to come back to the country as mentioned by one of the returnees:

“*Return was my original intention and my migration was never permanent*”*.*

Some prerequisites for permanent return were stressed upon by the majority of returnees and were referred to by one of them as ‘basic needs’. They included securing accommodation and, specifically, house ownership, schools for children and financial security in terms of enough savings before their return.

### Barriers to final return

Poor work environment, insufficient financial compensations, unsecured accommodation, and their children’s education were the main barriers to final return. Other reported barriers included high returnee expectations, which referred to their positioning in a job that matches their capacities and their ability to adapt to the work environment, healthcare system, policies, regulations and guidelines. This was in addition to peer obstructions and hostility as reported by some of the returnees.

### Recruitment and workplace of the permanently returned physicians

Personal relations and networks were the main route for recruitment for almost all returnees in both the public and private sectors, as illustrated by the following returnees’ quotes:

“*I got offers while I was still outside through friends and personal contacts and they were from public and private sectors*”.

“*I kept my relations which have helped me to find my current job when I came back*”.

Private part-time work was common for many physicians who permanently returned to the country. Full-time private work was limited to contracts with specific private institutions, but dual practice in public and private, with a dominance of private work, was the norm in most cases.

Three main aspects were stated as contributing to preferring employment in the private sector, namely financial reasons, since private sector compensations were higher, the concentration of private work being in Khartoum (the capital city), and the work environment, since the private sector was seen as “*more organized and easy to work in*”.

### Returnees’ opinions on options to improve medical diaspora contributions

Most of the suggestions made by the study participants to improve medical diaspora contributions focused on the issue of coordination of contributions. The majority of stakeholders considered short-term contributions as the most appropriate option for Sudan. However, supporting return migration through appropriate incentives targeting barriers to return was also stressed upon. Furthermore, encouraging expatriates large-scale investments in the healthcare sector was suggested in collaboration with the private sector.

## Discussion

The contributions of the Sudanese medical diaspora were found to be of a small magnitude, infrequent and not well organized. Nevertheless, their willingness to contribute to the improvement of the HCDS in Sudan was evident. This supports the findings from the study by Ibrahim and Bidwell in 2011 [18], reporting that over 90 % of Sudanese physicians working in the Republic of Ireland were willing to contribute to the healthcare system in Sudan, providing inputs in knowledge and skills transfer and in building useful institutional links with overseas institutions. Examples of these are (1) the Memorandum of Understanding signed between the Academy of Medical Royal Colleges in the United Kingdom and the Sudan Medical Specialization Board, which was facilitated by the Sudan Doctors Union in United Kingdom and the Republic of Ireland [44] and (2) the Irish-Sudanese Immigrant Physicians Training Initiative. A proposal for institutional twinning and collaboration between St James’s Hospital in Dublin, Ireland, and different Sudanese tertiary hospitals and centres, was proposed to be organized by the Sudan Medical Association UK and Ireland [45], in addition to their technical support to a number of decentralized localities in the provision of healthcare and service delivery [46].

A growing body of evidence is suggesting skilled professionals in diasporas are a considerable source of knowledge facilitated by countries networks [47]. Involvement of expatriate professionals in different country programmes through short visits and assignments is encouraged by the International Organization for Migration, which has organized an initiative called ‘Migration for Development’ in Africa - this Ghana/Netherlands programme is one such example that is quoted for its success [3, 5]. However, the sustainability of such programmes in the long run is challenged for two main reasons. First, such initiatives depend on the goodwill of emigrants and may prove difficult to sustain in the long term without proper facilitation from receiving countries. Second, many countries affected by migration of healthcare professionals are not able to meet the cost of such programmes, which include those of transport, accommodation and subsistence allowances to the participating individuals. Although donor support may be sought, programmes that are dependent on external funding prove difficult to sustain in the long term [48, 49].

Another area of diaspora engagement that has emerged over the past two decades is virtual participation [50, 51]. This form of long-distance participation of migrants has been promoted by advances in information and communication technologies which allow the utilization of the professional expertise of the diaspora from their overseas bases. While this is an attractive option, the lack of proper communication infrastructure might limit its success in developing countries such as Sudan [27, 50, 51].

The World Health Organization Global Code of Practice on the International Recruitment of Health Personnel has advised member states to facilitate the transfer of knowledge and skills of migrants to the best benefit of both source and destination countries, which could be achieved through proper organization of diaspora contributions and involvement of stakeholders to minimize the costs of diaspora contributions on developing countries [52]. Developing countries are encouraged to create innovative systems to foster transfer of knowledge. Organizing short-term returns of expatriates and supporting their sabbatical stays in the country to conduct short assignments such as training, is a good measure to promote knowledge and skills exchange [53].

Organization of diaspora contribution initiatives is the main challenge facing the medical diaspora in Sudan. This study has revealed that the main barriers facing diaspora contributions are organizational. Inappropriate organization and coordination was reported from the receiving local institutions and was attributed to the absence of one assigned body to deal with medical diaspora contributions to the HCDS in Sudan. This is in addition to the reported duplications and waste of resources, as one local institution may be approached by more than one diaspora association at a time since most of contributions are through personal initiatives and organized through personal relations and links.

Generally, the extent of permanent return is not properly documented. The number of professionals who returned to the country was recorded to be high in the years following the signature of the Comprehensive Peace Agreement in 2005 between North and South Sudan, which was seen then to have been enhanced by the expected economic growth in the country [54]. However, following the secession of the South in July 2011 the return movements decreased significantly [55].

The movement of healthcare workers depends mainly on personal values, taking into account the social, political and economic factors in both donor and recipient countries [56]. The findings of this study reveal that 31 % of the study participants were willing to permanently return to Sudan within the following 5 years. Available evidence shows that return is more likely to be beneficial when it occurs after a moderately long spell abroad of roughly 5–10 years [57]. Such conditions commonly allow migrants to save sufficient resources to ease their reinsertion into the home society [27]. Despite the desire to return to the home country being considered by the United States Department of State as a feature of people in diasporas, it is not necessarily a commitment to do so [58]. A primary intention to return was found to be the main motivation to a permanent return, as most of the physicians made their migration decision on the basis of short-term emigration. Offspring higher education was considered the primary reason for return, especially for migrants in the Gulf States and Saudi Arabia compared to those in the United Kingdom, Republic of Ireland, and the United States, since it is easier for migrants to obtain citizenship and access university education for their children in the latter [54]. Nevertheless, medical returnees often linked the financial constrains associated with a return to Sudan with the costs of education for their offspring. The challenges posed by the process of equivalence of foreign higher school certificates with the Sudanese certificates results in reduction of the overall grades to get accepted in public universities, forcing most parents to send their children to private education, which is costly [54]. Further, relatively higher public university tuition fees are charged to those who return, thus causing returnees to re-migrate to fulfil their offspring’s educational needs [59]. Some higher education policies have targeted the problem of returnees’ offspring education, especially for those who are working in public academic institutions, allowing acceptance with major discounts in the academic fees. Additionally, these policies exempt them from fulfilling some academic requirements for acceptance in various study disciplines. The newly developed higher education policies that permit academic staff from diaspora to have multiple contracts in many public and private universities on a part-time basis have been reported to encourage return migration to seek employment in public academic institutions [54].

Another reported facilitating policy was the dual residency card issued by the Ministry of Interior to expatriates holding other nationalities. Dual or multiple citizenship and residency rights enhance diaspora contributions to their home countries by making travel and investment easier [60]. They also benefit sending countries whose migrants adopt host country’s citizenship, which in turn promotes their integration in the host country and improves their earnings and job opportunities, and thus their contribution to their countries of origin [61]. A rise in expatriates’ contribution to sending countries that have allowed dual citizenship was documented in many countries including Ecuador, Costa Rica, Dominican Republic, Colombia, and Brazil [62].

Finding a job with the experience and specialized qualification requirements to meet returnees’ expectations is a problem reported by most of the returnees. Despite the fact that return migration in Sudan is documented to have brought important knowledge and skills, this was not utilized properly [63]. It was argued by Oxfeld and Long [64] that, even though returnees still face social and professional difficulties on their return, the contacts they have maintained and their back-and-forth movements reduce these difficulties. Indeed, medical returnees are recruited in Sudan depending mainly on the personal relations, contacts and links that they maintained while abroad. However, this affected their professional integration within the system and created conflicts with their peers, described by many as peer obstruction. Additionally, this has led to the inefficient utilization of the gained experiences, knowledge and skills to the maximum benefits of the HCDS, since many returnees were employed in the private sector in the capital city and large urban areas.

The findings of this study showed that 83 % of respondents managed to earn additional academic/professional qualifications while abroad, which in turn increases their expectations to be positioned in leading or highly paid positions. Returnees’ high expectations were suggested by many as a barrier for integration within the healthcare system in Sudan. However, according to Ministry of Labour records, few returnees were placed in leading positions and many had to compete for middle positions, which were reserved for the local staff as part of career progression [63], which made their integration more difficult.

The problems associated with return migration have led to a shift in focus to means for engaging medical diasporas that do not involve permanent return. Diaspora engagement initiatives have emerged as a leading policy option to meet the skill gaps created by the large-scale departure of skilled professionals in developing countries [27]. Tapping the MDOs to reduce effects of medical migration on the healthcare system in Sudan was recommended by studies which appraised the MDOs against other migration management strategies, such as staff retention strategies and introduction of a mid-level substitution cadre, suggesting MDOs as a financially and legally feasible and effective strategy for Sudan [3].

Options identified by this study to improve medical diaspora contributions to the development of the HCDS in Sudan have addressed several areas identified previously as barriers to diaspora contributions. As short-term return was found to be the most appropriate option to Sudan, effective coordination of diaspora contribution initiatives through the establishment of a defined body from within the healthcare sector to liaise with the medical diaspora and organize diaspora contributions was identified by this study as the main option for achieving effective, efficient and sustainable contributions to HCDS in Sudan. Countries like Eritrea, India and Brazil have established institutions whose main role is to build relationships with the diaspora [65].

Some options have targeted the return migration through measures to facilitate returnee integration in the healthcare system such as setting of standards and criteria for recruitment and transparency in job selection within the healthcare system. Others have targeted their integration in the country in general through building collaborative relationships with stakeholders such as the Ministry of Higher Education. Other measures argued to produce goodwill relationships that encourage the diaspora investment in the home country were proposed in terms of joint investment and collaboration with the private sector. Investors from expatriates are expected to be more loyal than regular investors in times of hardship and their interest in financing infrastructure in health and education projects is documented [66]. Such measures were reported to have increased diaspora contributions in many countries including the Philippines, India and several African countries [27, 67].

## Conclusions

The magnitude of the current contribution initiatives does not proportionately match the size of Sudanese medical diasporas in the five areas of the study; yet, their willingness to contribute is remarkable. Short-term contributions were the focus of this study since permanent return is becoming rare due to the country’s current situation. This study has revealed that the main barriers facing diaspora contributions are organizational. Creation of a coordinating body from within the healthcare sector in Sudan to advocate the positive role of the medical diaspora in the development of the healthcare system, to document the knowledge and skill gaps that are needed to be filled in the HCDS in Sudan, to liaise with the medical diaspora, and to effectively coordinate their contributions is the proposed mechanism through which to improve diaspora contributions to the HCDS in Sudan.

## Abbreviations

HCDS: healthcare delivery system
MDO: medial diaspora options

## Footnotes

1 HCDS comprises different organizations that contribute directly (i.e. service provision) or indirectly (i.e. health workforce production) to the delivery of healthcare services to the people [11].
